# Dietary inflammatory potential and the incidence of depression and anxiety: a meta-analysis

**DOI:** 10.1186/s41043-022-00303-z

**Published:** 2022-05-28

**Authors:** Xiaoling Li, Meicui Chen, Zhicui Yao, Tianfeng Zhang, Zengning Li

**Affiliations:** Department of Nutrition, Hebei Province Key Laboratory of Nutrition and Health, The First Hospital of Hebei Medical University, Shijiazhuang, 050031 Hebei Province China

**Keywords:** Depression, Anxiety, Dietary inflammatory potential, Psychological health

## Abstract

The potential modifiable role of diet in common psychological disorders, including depression and anxiety, has attracted growing interest. Diet may influence the occurrence of mental disorders through its inflammatory characteristics. The purpose of this meta-analysis was to explore whether dietary inflammatory potential is associated with the risk of depression and anxiety. A systematic literature search was conducted in PubMed, Web of Science, and Embase databases up to February 2021. Articles related to dietary inflammatory potential and risk of depression or anxiety were included. After the elimination of repetitive and irrelevant literature, we conducted quality assessment, publication bias, and sensitivity analysis. In total, 17 studies with a total of 157,409 participants were included in the final analysis. Compared with the lowest inflammatory diet group, the highest group was significantly associated with the incidence of depression and anxiety, with the following pooled odds ratios (ORs) and 95% confidence intervals (95% CIs): 1.45 (1.30 ~ 1.62) for depression and 1.66 (1.41 ~ 1.96) for anxiety. A subgroup analysis by gender showed that this association was more prominent in women. For depression, the increased risk was 49% in women (OR 1.49, 95% CI 1.28 ~ 1.74) and 27% in men (OR 1.27, 95% CI 1.06 ~ 1.52). As for anxiety, the increased risk was 80% in women (OR 1.80, 95% CI 1.30 ~ 2.49) and 47% in men (OR 1.53, 95% CI 0.81 ~ 2.89). As a result, long-term anti-inflammatory eating patterns may prevent depression and anxiety, whereas pro-inflammatory eating patterns may promote these conditions. People should add more fish, fish oil, fresh fruit, walnuts, and brown rice to their diet.

**Supplementary Information:**

The online version contains supplementary material available at 10.1186/s41043-022-00303-z.

## Introduction

Mental health disorders represent a major public health concern. More than 300 million people suffer from depression worldwide, and more than 70% of people experience anxiety in their lifetimes. Psychiatric disorders impose great societal burden on developed countries [1]. According to the World Health Organization, depressive and anxiety disorders cost the global economy $1 trillion in lost productivity each year [2]. Identifying modifiable risk factors is of potential value to intervention strategies for preventing mood disorders and decreasing their severity.

Among the modifiable factors, diet is one of the main lifestyle-related factors for mental disorders that individuals are exposed to daily. It is worth noting that experts from the International Society for Nutritional Psychiatry Research state that “diet and nutrition are the central determinants of mental health” [3]. Recently, diet and circulating inflammation markers have been reported to be major contributors to the incidence of depression [4, 5]. Diet affects the levels of inflammatory factors, such as IL-1*β*, IL-6, IL-10, and TNF-*α*, by regulating the immune system, intestinal flora, and macrophages. A diet that can raise the level of inflammation in the body is called pro-inflammatory diet, and a diet that has an opposite effect is called an anti-inflammatory diet. To understand the inflammatory potential of diet, experts have developed the Dietary Inflammation Index (DII) to assess the inflammatory capacity of diet according to the pro-inflammatory and anti-inflammatory efficacy of different dietary components [6]. A low DII score indicates anti-inflammatory potential, whereas a high DII score reflects pro-inflammatory potential. Epidemiological studies have explored the association between dietary inflammatory potential and mental health disorders. Some reported increased risk associated with a pro-inflammatory diet, but others reported no association [7, 8].

The inconsistent findings of previous research and the lack of overview of other major mental health outcomes, such as anxiety, make it difficult to draw a reliable and universal conclusion. Therefore, the purpose of the present meta-analysis was to summarize the evidence on the association between the inflammatory potential of diet and depression or anxiety.

## Methods

This meta-analysis was conducted in accordance with the guidelines indicated in the Preferred Reporting Items for Systematic Reviews and Meta-Analyses (PRISMA) protocols [9]. The PRISMA checklist is shown in Additional file 1: Table S1.

### Definition and diagnosis of depression and anxiety

Depressive disorder, a mood disorder caused by multiple causes, has a variety of unique symptom combinations. This disorder is characterized by persistent low mood, loss of interest, difficulty in concentrating, sleep disturbance, and fatigue, and its severe form is characterized by functional impairment and suicidal tendency [10]. The main diagnostic methods for depression are scales and questionnaires, including the Center for Epidemiologic Studies Depression Scale (CES-D), Patient Health Questionnaire (PHQ) [11], Hospital Anxiety and Depression Scale (HADS) [12], and so on.

Anxiety is an unpleasant emotional experience of inner tension, fear, or anticipation of an adverse situation, and coping with it is difficult. Anxiety disorder can increase the risk of depression. The HADS is a common diagnostic tool in clinics.

### Search strategy

A systematic literature search was performed in PubMed, Web of Science, and Embase from inception to February 2021 for the identification of relevant studies. The following search terms were used: (“dietary inflammatory potential” or “inflammatory diet” or “diet-related inflammation” or “pro-inflammatory diet” or “anti-inflammatory diet” or “inflammatory potential of diet” or “dietary pattern” or “DII” or “dietary inflammatory index” or “dietary inflammatory score”) and (“depression” or “anxiety” or “psychological disease” or “melancholia” or “depressive disorder” or “anxiety disorder”). Searches were limited to articles written in English. We also searched the references of related articles to obtain other potential studies. Two researchers (XLL and ZCY) searched independently with the same retrieval strategy. According to the inclusion and exclusion criteria, the included studies were screened out by eliminating duplicate studies, reading the titles and abstracts of articles, and reading full texts. Disagreements were resolved by an experienced third researcher (ZNL).

### Inclusion and exclusion criteria

The following inclusion criteria were used: (1) population: adults; (2) intervention/comparison: dietary inflammatory index or blood inflammatory markers; (3) outcomes: clinical depression or anxiety assessed by the study staff or medical records; depressive symptoms or anxiety assessed by validated scales or questionnaires, and defined according to the validated cutoffs of these scales; the use of anti-depressive drugs considered only when it was combined to clinical depression or depressive symptoms assessment; (4) Study design: observational studies (cohort, case–control, or cross-sectional studies). Exclusion criteria included: (1) population: non-adults; (2) intervention/comparison: not measured the inflammatory potential of the diet; (3) outcomes: any other outcome outside of the inclusion criteria; (4) study design: non-observational studies; (5) review articles, studies not published as a full article (e.g., conference abstracts), and studies lacking sufficient data.

### Data extraction

Data and characteristics extracted from each study included: first author’s surname, year of publication, geographical location, study design, sample size, percentage of females, mean age, length of study follow-up (if applicable), diagnosis of depression or anxiety, assessment tool of dietary inflammatory potential, risk estimates, the 95% CIs from the most fully adjusted models for the association between a pro-inflammatory diet and the incidence of depression or anxiety, and adjustment factors. Since the majority of articles reported gender-specific effects, we extracted separate effect sizes for males and females.

Subjects were grouped differently in the included studies according to dietary inflammatory potential. Where studies had stratified subjects into groups (tertiles, quartiles, and quintiles), the pro-inflammatory diet was defined as the highest grouping and the anti-inflammatory diet was defined as the lowest grouping. The likelihood of depression or anxiety was obtained from a combination of hazard ratio (HR), odds ratio (OR), and relative risk (RR) effects.

### Quality assessment

Quality of cohort and case–control studies was assessed using the Newcastle–Ottawa Quality Assessment Scale (NOS) [13], and quality of cross-sectional studies was assessed using the Agency for Healthcare Research and Quality (AHRQ) scale [14].

The total NOS score was nine points. Studies identified as having the NOS ≥ 7 are considered high quality, whereas studies with a total NOS score < 7 are considered low quality. AHRQ includes 11 items. An item would be scored “0” if it was answered “NO” or “UNCLEAR”; if it was answered “YES,” then the item scored “1.” Article quality was assessed as follows: low quality = 0–3; moderate quality = 4–7; high quality = 8–11.

### Statistical analysis

In the meta-analysis, the combined OR value and 95%CI were used to evaluate the relationship between dietary inflammation and the risk of depression and anxiety (the likelihood of depression or anxiety in the highest inflammatory diet group, compared with the lowest inflammatory diet group). The associated effects include the OR, RR, and HR. Considering that depression with a low incidence rate, the OR, HR, and RR can be directly combined with the effect sizes and expressed as OR. The most fully adjusted HRs and RRs with their 95% CIs from individual studies were extracted and transformed into their logarithms for the stabilization of variances and normalization of distributions.

We used the I^2^ metric to quantify the heterogeneity between studies. *I*^2^ > 50% indicated high heterogeneity, *I*^2^ = 25–50% indicated moderate heterogeneity, and *I*^2^ < 25% indicated low heterogeneity [15]. A random-effects model was applied when *I*^2^ statistic > 50%, or a fixed-effects model was used otherwise. As a result of the heterogeneity of the results, the following factors were considered for subgroup analysis based on professional knowledge and previous reports in the historical studies: sex, study design, effect measure, average age at baseline, follow-up period, sample size, and diagnostic method.

We conducted a sensitivity analysis in which the meta-analysis was repeatedly carried out after omitting each study in turn to observe the stability of the comprehensive results [16]. The potential publication bias was graphically represented by funnel plots, and the funnel plot asymmetry was evaluated with Egger’s linear regression test and Begg’s test [17, 18]. All statistical analyses were performed using STATA version 15. *P* values were considered significant at a level of < 0.05.

## Results

### Study selection

**Fig. 1:**
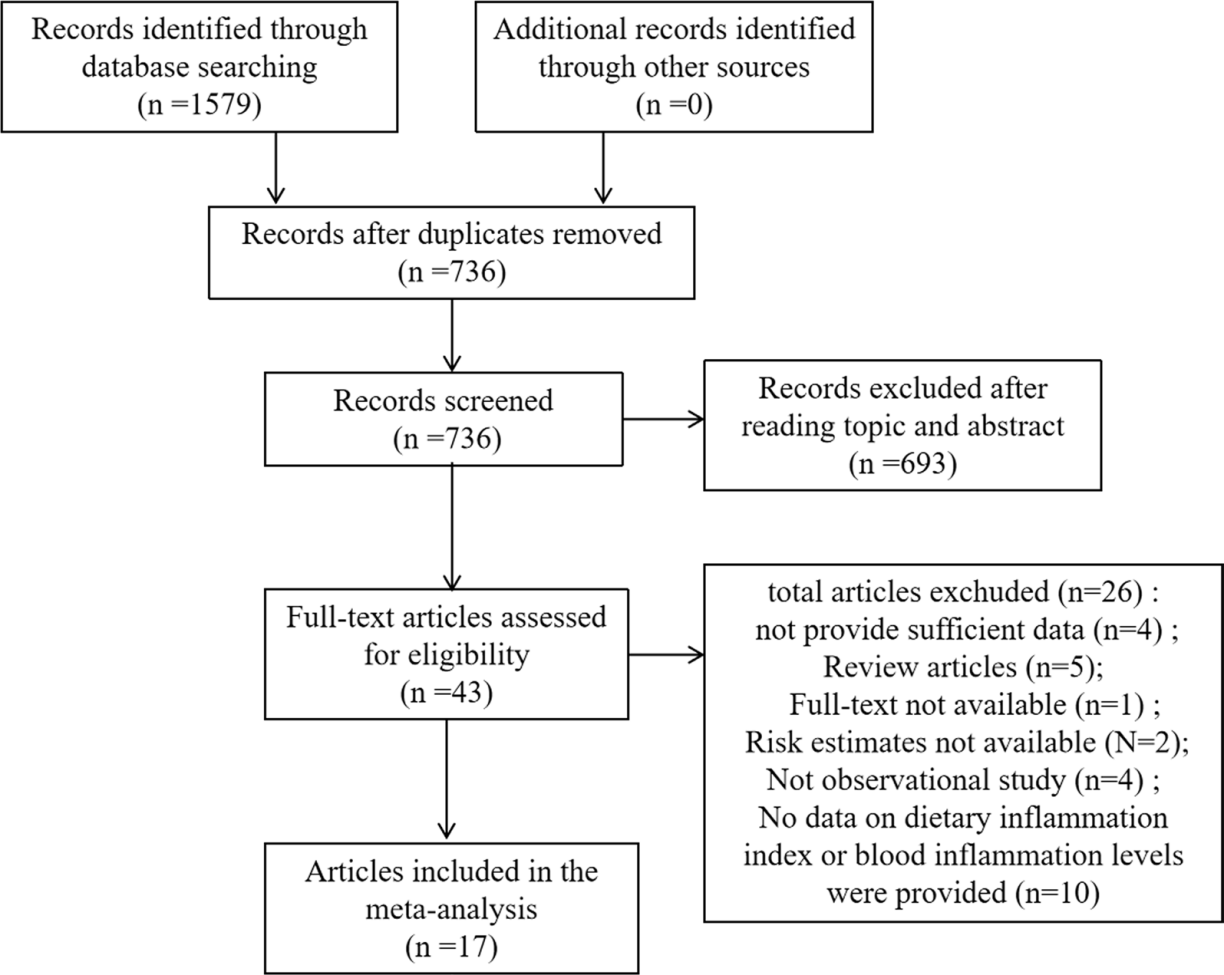
Flowchart demonstrating the search strategy for the meta-analysis

According to the search strategy, 1579 studies were collected, including 695 PubMed, 729 Embase, and 155 Web of Science. After initial assessment, the remaining 736 papers were included. After reading the title and abstract, 43 articles were retained. Twenty-six publications were excluded after full-text screening for different reasons (Additional file 2: Table S2). The final selection yielded 17 articles to be included for analysis [19–35]. The detailed selection process of selected studies is shown in Fig. 1.

### Quality assessment

The mean quality score was 7.7 assessed by the NOS for cohort studies and 7.4 by the AHRQ for cross-sectional studies (Additional file 3: Tables S3a,b).

### Study characteristics

A total of 157,409 subjects from eight prospective cohort studies [20, 21, 25, 29–32, 34] and nine cross-sectional studies [19, 22–24, 26–28, 33, 35] were included. Seventeen studies reported the effect of dietary inflammatory potential on depression, and four of these studies reported its effect on anxiety [22, 26–28]. Most studies involved both men and women, whereas four studies included women only [19, 25, 31, 32]. The basic characteristics and quality evaluation results of studies are shown in Table 1.

**Table 1:** Characteristics of studies included in the meta-analysis

Study	Location (cohort)	Design	Follow-up, years	Subjects at baseline, n	Females, %	Age at baseline	Quality score
Açik et al. [19]	Turkish	Cross-sectional	N/A	134	100	24 ± 5.1	7
Adjibade et al. [20]	French	Cohort	5.4	26,730	24	48.3 ± 11.2	9
Akbaraly et al. [21]	UK	Cohort	5	4246	25	60.9 ± 5.9	7
Bergmans et al. [22]	USA	Cross-sectional	N/A	11,592	52	48.1 ± 7.8	7
Haghighatdoost et al. [23]	Iran	Cross-sectional	N/A	3363	59	36.2 ± 9.2	8
Jorgensen et al. [24]	USA	Cross-sectional	N/A	11,624	48	46.0 ± 0.34	7
Lucas et al. [25]	USA	Cohort	12	43,685	100	62.6 ± 7.0	8
Phillips et al. [26]	Ireland	Cross-sectional	N/A	1992	51	59.7 ± 5.5	8
Salari-Moghaddam et al. [27]	Iran	Cross-sectional	N/A	2016	59	35.7 ± 5.3	7
Salari-Moghaddam et al. [28]	Iran	Cross-sectional	N/A	3363	58	36.3 ± 7.8	7
Sanchez-Villages et al. [29]	Spain	Cohort	8.5	15,093	59	38.3 ± 12.1	7
Shivappa et al. [30]	Iran	Cross-sectional	N/A	300	100	16.2 ± 1.0	8
Shivappa et al. [31]	Australia	Cohort	12	6438	100	52.0 ± 1.4	8
Shivappa et al. [32]	USA	Cohort	8	3608	57	61.4 ± 9.2	8
Vermeulen et al. [33]	Italy	Cohort	9	827	58	73.8 ± 6.8	8
Wirth et al. [34]	USA	Cross-sectional	N/A	18,875	51	46.9	8
Adjibade et al. [35]	France	Cohort	12.6	3523	58	49.5 ± 6.2	7

The majority of these studies focused on gender-specific effects, whereas mixed sex effects were reported by four papers only [22, 24, 33, 34]. The diagnostic criteria for depression and anxiety, the dietary inflammation assessment scheme, the food parameters derived (the total number of dietary components investigated when a dietary survey is conducted), and the model adjustments used for each study can be found in Table 2. The studies mostly assessed dietary inflammatory potential with the DII or modified DII (E-DII, ADII, and FDII), whereas two studies assessed the potential by measuring blood cytokine level [25, 34]. Two studies [25, 29] diagnosed depression with either self-raised physician consultation or antidepressant use, and the other studies measured depression and anxiety disorders with various methods, including the CES-D [20, 21, 26, 30–32, 34], PHQ-9 [22, 24, 35], HADS [23, 26–28], Depression Anxiety and Stress Scales (DASS-21) [33], and Zung Self-Rating Depression Scale [19]. We performed a subgroup analysis of different diagnostic methods (Table 3).

**Table 2:** Study-specific case definition, methods of inflammatory diet assessment, and effect size model adjustments

Study	Case definition	Criteria for case	Assessment of Inflammatory diet	Food parameters derived	Model adjustments
Lucas et al. [25]	Depression	Self-reported physician-diagnosed depression and regular antidepressant use (strict definition)	Measured CRP, IL-6, and TNF-a receptor 2	39	Age, BMI, total energy intake, smoking, physical activity, menopause status, HRT, marital status, retired, education, husband education, ethnicity, multivitamin use, reported diagnosis of cancer, high blood pressure, hypercholesterolemia, heart disease, diabetes, MHI-5 score at baseline, alcohol intake, caffeine intake
Sanchez-Villages et al. [29]	Depression	Use of antidepressants and/or physician diagnosis	DII	28	Age, BMI, smoking, physical activity during leisure time, use of vitamin supplements, total energy intake, presence of diseases at baseline (CVD, diabetes, hypertension, and dyslipidemia)
Akbaraly et al. [21]	Recurrent depressive symptoms	CES-D score ≥ 16 or treated by antidepressants	DII	27	Age, ethnicity, total energy intake, socioeconomic status, marital status, smoking habits, physical activity, alcohol intake, coronary heart diseases, type 2 diabetes, hypertension, HDL cholesterol, use of lipid-lowering drugs, central obesity, cognitive impairment
Shivappa et al. [31]	Depressive symptoms	CES-D-10 score ≥ 10	DII	26	Total energy intake, highest qualification completed, marital status, menopause status, night sweats, major personal illness or injury, smoking, physical activity, BMI, depression diagnosis or treatment
Wirth et al. [34]	Depressive symptoms	PHQ-9 score ≥ 10	DII	26	Race, education, marital status, perceived health, current infection status, family history of smoking, smoking status, past cancer diagnosis, arthritis, age, average nightly sleep duration
Bergmans et al. [22]	Depression	PHQ-9 score ≥ 10	DII	N/A?	Age, ethnicity, poverty income ratio category, employment status, health insurance status, educational status, marital status, BMI, smoking, physical activity, sedentary time, use of vitamin supplements, total energy intake, menopause (among women), any comorbidity (history of hypertension, dyslipidemia, diabetes, CVD, respiratory illness, or cancer)
	Frequent anxiety	HRQOL: Frequent anxiety was defined as reporting feeling worried, tense, or anxious > 14 days out of the past 30			
Adjibade et al. [35]	Depressive symptoms	CES-D (French) score ≥ 17 in men and ≥ 23 in women	DII	36	Age, intervention group during trial phase, education level, marital status, socio-professional status, energy intake without alcohol, number of 24-h dietary records, interval between the 2 CES-D measurements, smoking status, physical activity, BMI, cancer or cardiovascular events during follow-up
Phillips et al. [26]	Depressive symptoms	CES-D score ≥ 16	E-DII	26	Age, BMI, physical activity, smoking, alcohol consumption, antidepressant use, history of depression
	Anxiety	HADS scores ≥ 13			
Jorgensen et al. [24]	Depression	PHQ-9 score ≥ 10	DII	28	Race, education, annual household income, use of prescription cholesterol-lowering medication, lifetime history of cancer, BMI, physical activity, age, sex, current smoking status, taking dietary supplements in past 30 days, total energy intake
Salari-Moghaddam et al. [27]	Depression	HADS ≥ 8	DII	29	Age, sex, total energy intake, marital status education, family size, home ownership, antidepressant use, vitamin supplements use, smoking physical activity
	Anxiety	HADS ≥ 8			
Shivappa et al. [30]	Depressive symptoms	CES-D score ≥ 16	DII	24	Age, sex, race, BMI, education, smoking habits, yearly income, CES-D at baseline, statins use, NSAIDs or cortisone use
Shivappa et al. [32]	Depressive symptoms	DASS-21 (Persian) score > 9	DII	31	Age, energy, physical activity, BMI, smoking, presence of chronic disease, diet supplement use, salary, marital status
Vermeulen et al. [33]	Depression	CED-D score ≥ 20	Measured CRP, IL6 and TNF- a	10	Sex, age, marital status, education in years, depressive symptoms at baseline, smoking status, physical activity, antidepressant use, anti-inflammatory drugs, cardiovascular events, diabetes, waist circumference
Haghighatdoost et al. [23]	Depression	GHQ scores ≥ 12	DII	27	Age, marital status, education, BMI, smoking, physical activity, anti-psychotropic medicine use, suffering from gastrointestinal disorders
Adjibade et al. [20]	Depressive symptoms	CES-D ≥ 17 for men and ≥ 23 for women	ADII	34	Age, sex, marital status, educational, occupational categories, monthly household income, residential area, energy intake without alcohol, number of 24-h dietary records, alcohol intake, smoking status, physical activity, BMI, cancer, type 2 diabetes, and cardiovascular events
Açik et al. [19]	Depression	Zung Self-Rating Depression Scale ≥ 50	DII	29	Age, total energy intake, ethnicity, smoking, alcohol consumption, physical activity, minimally active, BMI and energy intake
Salari-Moghaddam et al. [28]	Depression	HADS ≥ 8	FDII	28	Age, sex, total energy intake, marital status education, family size, home ownership, antidepressant use, vitamin supplements use, smoking physical activity
	Anxiety	HADS ≥ 8			

Key: *TNF-a*  tumor necrosis factor-alpha, *CRP*  C-reactive protein; *IL-6*  interleukin-6, *BMI*   body mass index, *HRT*   hormone replacement therapy, *MHI-5*   mental health inventory, *DII*   Dietary Inflammatory Index, *CES-D*   Center for Epidemiologic Studies Depression Scale, *PHQ-9*   Patient Health Questionnaire, *HDL*   high-density lipoproteins, *PASE*   Physical Activity Scale for the Elderly, *DASS-21*   Depression Anxiety and Stress Scale, *NSAID*   nonsteroidal anti-inflammatory drug, *IADL*   Lawton Instrumental Activities of Daily Living, *HADS* Hospital Anxiety and Depression Scale, *GHQ-12*   General Health Questionnaire, *DII*   Dietary Inflammation Index, *E-DII*   energy-adjusted DII, *ADII*  Alternate Dietary inflammatory Index, *FDII *  food-based dietary inflammatory index, *HRQOL*  Health-Related Quality of Life. Instrumental Activities of Daily Living, *HADS*  Hospital Anxiety and Depression Scale, *GHQ-12*   General Health Questionnaire, *DII * Dietary Inflammation Index

*E-DII*  energy-adjusted DII, *ADII*   Alternate Dietary inflammatory Index, *FDII*   food-based dietary inflammatory index, *HRQOL*   health-related quality of life

**Table 3:** Subgroup analysis

Subgroup factor	Subgroup	Studies	OR (95% CI)	Heterogeneity	*P*
				I^2^	
Study design	Cross-sectional	9 [19, 22–24, 26–28, 30, 34]	1.73 (1.53–1.95)	48%	0.02
	Cohort	8 [20, 21, 25, 29, 31–33, 35]	1.25 (1.16–1.35)	43%	0.06
Effect measure	RR	2 [25, 31]	1.33 (1.16–1.51)	32.2%	0.225
	HR	3 [29, 32, 35]	1.09 (1.05–1.54)	55.5%	0.081
	OR	12 [19–24, 26–28, 30, 33, 34]	1.30 (1.01–1.76)	86.7%	0.0002
Average age at baseline	< 50 years old	6 [21, 25, 26, 31–33]	1.29 (1.10–1.52)	52%	0.04
	≥ 50 years old	11 [19, 20, 22–24, 27–30, 34, 35]	1.50 (1.31–1.72)	62%	0.0003
Follow-up period (longitudinal only)	< 10 years	3 [25, 31, 35]	1.29 (1.05–1.58)	54%	0.09
	≥ 10 years	5 [20, 21, 29, 32, 33]	1.23 (1.12–1.35)	45%	0.08
Sample size	< 10,000	6 [20, 24, 25, 29, 34, 35]	1.30 (1.20–1.41)	40%	0.09
	≥ 10,000	11 [19, 21–23, 26–28, 30–33]	1.63 (1.35–1.96)	66%	0.0001
Diagnostic method	CES-D	7 [20, 21, 26, 30, 31, 33, 35]	1.19 (1.07–1.33)	9.6%	0.351
	PHQ-9	3 [22, 24, 34]	1.62 (1.05–2.48)	84.7%	0.001
	HADS	2[27, 28]	1.64 (1.30–2.08)	0.0%	0.383
	Treated by antidepressants	3 [21, 25, 29]	1.40 (1.24–1.57)	0.0%	0.422
	Other methods (Zung Self-Rating Depression Scale, GHQ, DASS-21)	4 [19, 23, 32]	1.05 (0.54–2.04)	53%	0.07

### Association between pro-inflammatory diet and depression

**Fig. 2:**
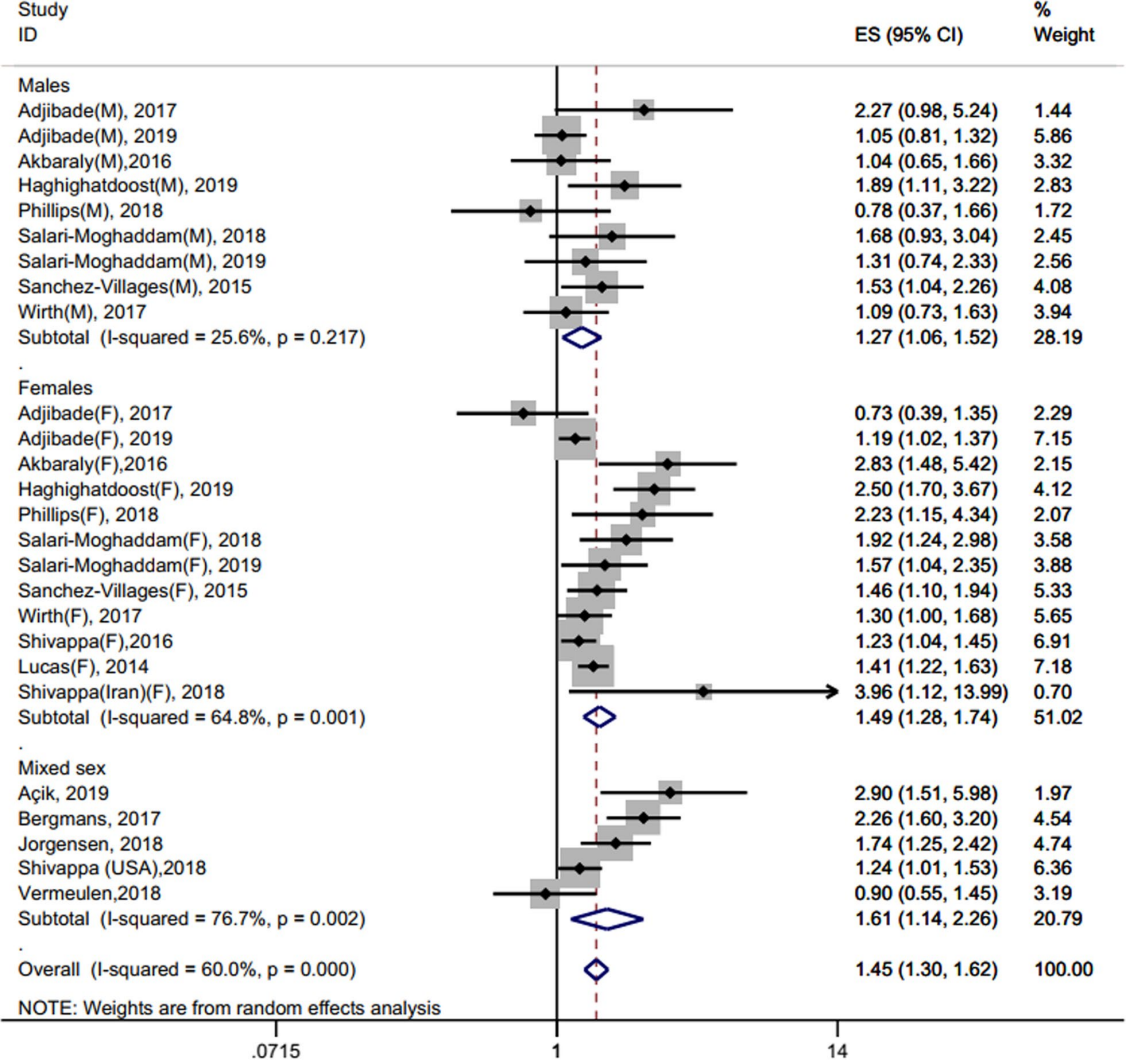
Random-effects meta-analysis and forest plot for the association between a pro-inflammatory diet and depression. Results are also subgrouped by sex-specific populations

We used a random-effects model to estimate the aggregate OR and 95% CI of all studies. The meta-analysis showed a positive correlation between the pro-inflammatory diet and the risk of depression and anxiety (pooled OR = 1.36, 95% CI = 1.18 ~ 1.56) with high heterogeneity (*I*^2^ = 78.2%, *P* < 0.0001). Seventeen studies reported the effect of dietary inflammatory potential on depression disorders in 26 populations (9 males, 12 females, and 5 mixed sex). The results of random-effects model showed that the risk of depression in pro-inflammatory diet group individuals were elevated when compared to those in anti-inflammatory diets (pooled OR = 1.45, 95% CI 1.30 ~ 1.62, *P* < 0.00001) (Fig. 2). Since the majority of studies reported separate effect sizes for males and females, results were also subgroups based on sex (Fig. 2). Effects were stronger in females (pooled OR = 1.49, 95% CI 1.28 ~ 1.74, *P* = 0.001), as opposed to males (pooled OR = 1.27, 95% CI 1.06 ~ 1.52, *P* = 0.001), and studies reporting mixed sex effects were significant (pooled OR = 1.61, 95% CI 1.14 ~ 2.26, *P* = 0.002).

Since some studies showed substantial heterogeneity, subgroup analysis was also conducted based on the following categorical variables: study design, effect measure, follow-up period (cohort studies only), sample size, average age at baseline, and diagnostic methods (Table 3). Subgroup analysis suggested that the source of potential study heterogeneity may be applicable to the average age at baseline, sample size, effect measure, and diagnostic method of the studies.

### Sensitivity analysis and publication bias to the analysis results of pro-inflammatory diet and depression

Sensitivity analysis showed that no study had significant influence on the overall results, indicating that the results were stable. Publication bias was examined using Begg’s rank correlation and Egger’s linear regression test. Egger’s test (*P* = 0.113) and Begg’s test (*P* = 0.142) were nonsignificant. The absence of publication bias was determined through funnel plot inspection (Fig. 4A). Little evidence of publication bias is evidenced by visual inspection of the plots.

### Association between pro-inflammatory diet and anxiety

**Fig. 3:**
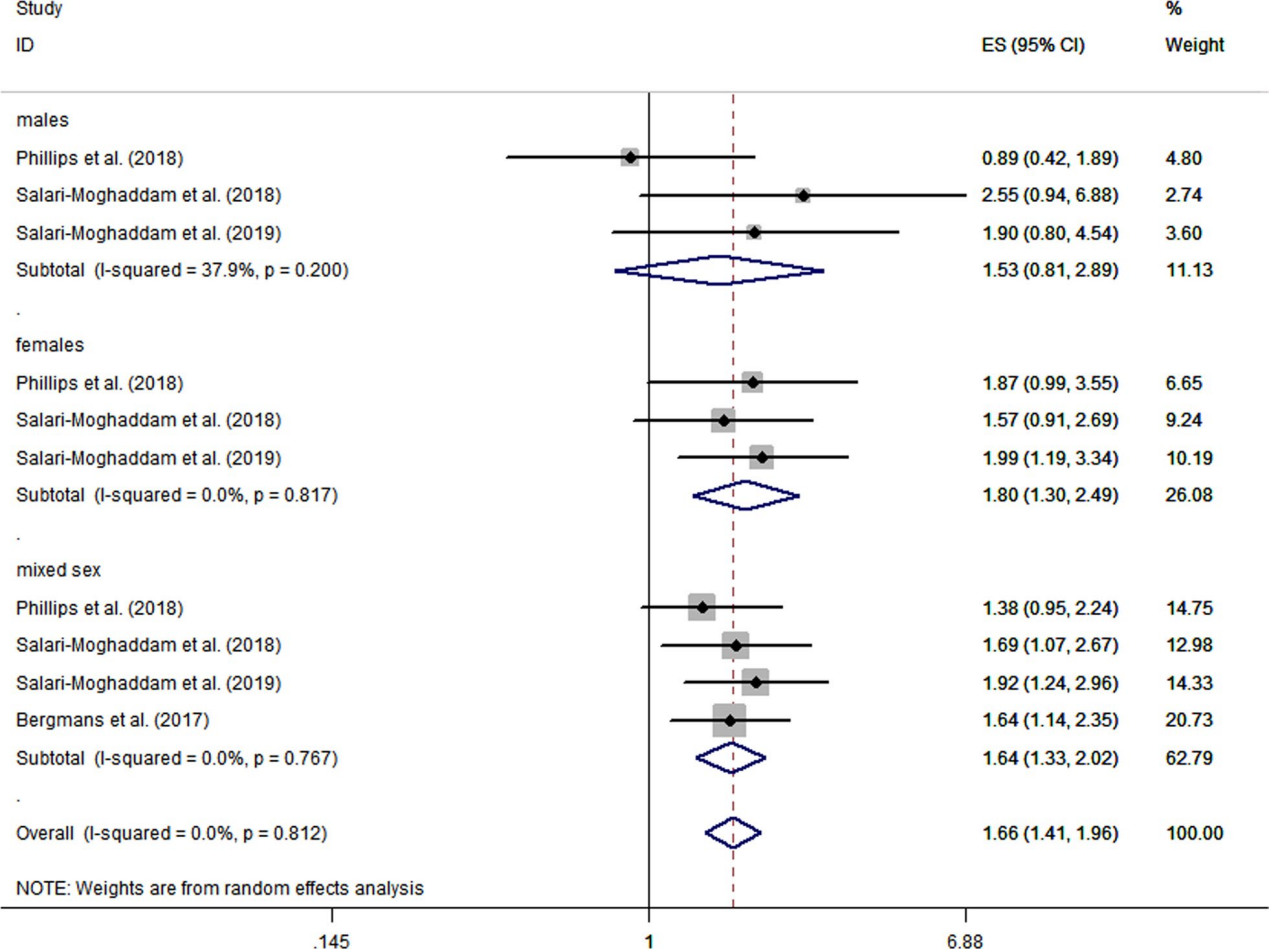
Random-effects meta-analysis and forest plot for the association between a pro-inflammatory diet and anxiety. Results are also subgrouped by sex-specific populations

The results of random-effects model showed that the risk of anxiety disorders in pro-inflammatory diet group individuals was elevated when compared to those in anti-inflammatory diets (pooled OR = 1.66; 95% CI 1.41 ~ 1.96, *P* < 0.0001), as shown in Fig. 3. Subgroup analysis based on gender showed that the influence of dietary inflammatory potential on the incidence risk of anxiety disorders was more significant in females (pooled OR = 1.80; 95% CI 1.30 ~ 2.49, *P* = 0.0003), as opposed to males (pooled OR = 1.53; 95% CI 0.81 ~ 2.89, *P* = 0.012).

### Sensitivity analyses and publication bias to the analysis results of pro-inflammatory diet and anxiety

**Fig. 4:**
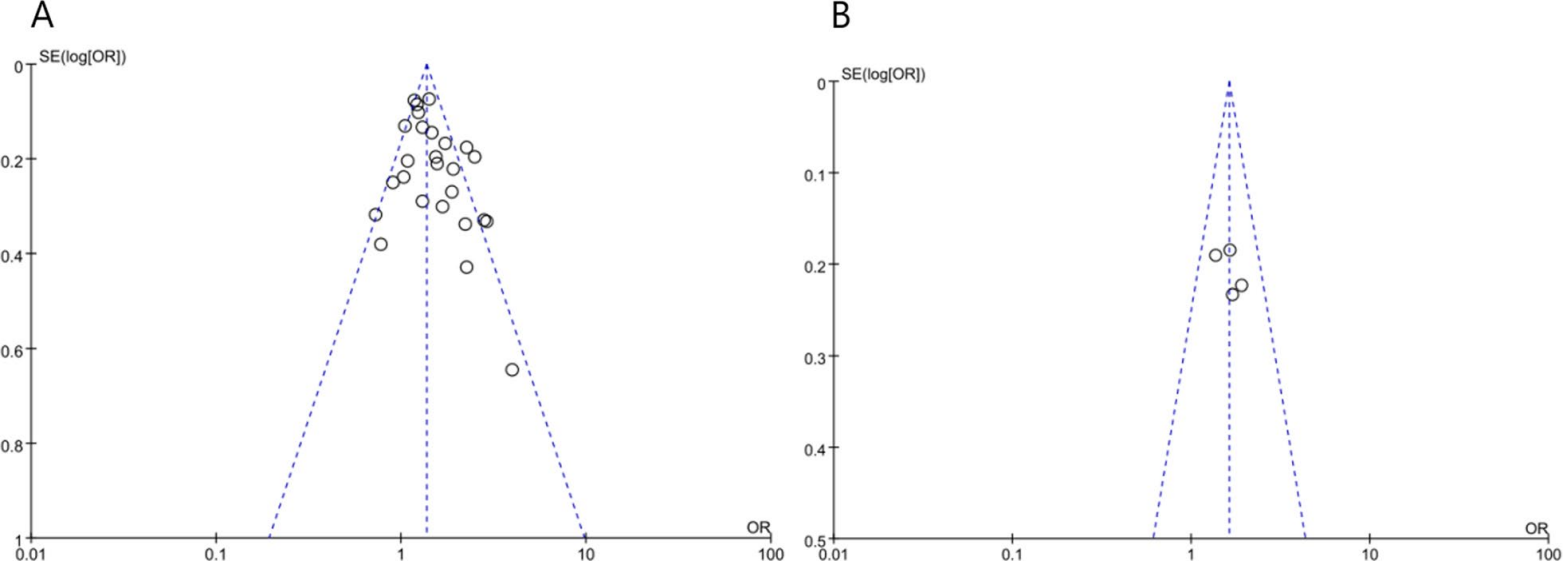
Funnel plot for the included study populations for the association between a pro-inflammatory diet with depression (**A**) and anxiety (**B**)

Removal of each individual study from the overall model demonstrated the robustness of the analysis. Sensitivity analysis showed that none of the studies had a significant impact on overall results. Both Egger’s test (*P* = 0.743) and Begg’s test (*P* = 0.602) did not identify any potential publication bia. Funnel plot indicated no publication bias (Fig. 4B).

## Discussion

Depression and anxiety disorders are the most common mental illnesses that damage human health. They seriously disrupt the lives and work of patients and impose heavy burden on families and society. Therefore, identifying the risk factors for depression and anxiety disorders is of great significance to their prevention. In recent years, the influence of diet factors on mental illness has been widely concerned.

The meta-analysis included 17 studies with 157,409 participants. The results showed that individuals on pro-inflammatory diets were 45% more likely to suffer from depression and 66% more likely to suffer from anxiety disorders than those on anti-inflammatory diets. These results are in agreement with a number of previous studies. Grooms et al. found that food ingredients with anti-inflammatory properties (curcumin, fiber, allicin) can prevent mental disorders [36, 37]. In a cross-sectional study performed by Miki et al., reduced incidence of depression was found among consumers of diets high in fruit and vegetable fiber [38]. The results from our analysis indicated a stronger association between the dietary sources of inflammation and the risk of developing depression and anxiety in females than in males. Although women’s mood and its susceptibility to dietary inflammatory potential have been proposed, further prospective cohort research is required to establish the relationship between depression and anxiety incidence and gender-specific diet inflammation effects.

The mechanisms by which dietary inflammatory potential correlates with the incidence of depression and anxiety are not fully clarified. Nevertheless, some studies showed that mental disorders are associated with circulating inflammation markers [39, 40]. Pro-inflammatory nutrients may activate the innate immune system that can lead to chronic low-grade inflammation [41, 42]. On the other hand, dietary factors can affect the markers of neuronal function [43, 44]. Anti-inflammatory diet, when combined with exercise, was found to up-regulate genes that contribute positively to neuronal plasticity in mice [45].

The DII, a novel tool for dietary inflammation potential assessment exploited recently, was employed for the included studies [46]. In 2013, Nitin Shivappa et al. developed and verified the revised DII with 45 dietary components and calculated the inflammatory effect index of each dietary component. A positive value represents the pro-inflammatory tendency of a diet, a negative value represents the anti-inflammatory tendency, and 0 represents neither pro-inflammatory nor anti-inflammatory tendency. DII is associated with various inflammatory markers, including C-reactive protein [47], interleukin-6, and homocysteine [48]. The high DII scores of dietary patterns (expressed as promoting inflammatory diet) are associated with the risk of many diseases, such as asthma [49], diabetes [50], obesity [51], and cancer [52].

Despite the strengths of the current analysis, there are certain limitations that should be noted. Firstly, different measurement methods and inclusion criteria adopted in the included studies may generate heterogeneity. For example, different scales and thresholds were used for the diagnosis of depression and anxiety. Despite this, we utilized random-effects models and subgroup analyses to limit and detect such sources of variability. Further, there are only four studies on anxiety; thus, more observational studies are needed to confirm this conclusion.

In addition, it shall seem plausible that dietary consumption preferences are influenced by an individual’s mental health status. Papier et al. investigated the effect of stress and selection for foods. Their results suggested that mild-to-moderate degree of stress could drive students toward three times higher consumption of processed food rather than choosing fruit and vegetables as found in unstressed students [53]. The role of reverse causation should therefore be considered in research.

In summary, the positive association between pro-inflammatory dietary style patterns with depression and anxiety found in this analysis can be useful in exploring strategies for the prevention and treatment of depression and anxiety. A scientific and healthy diet that includes a variety of anti-inflammatory food, such as fruit, vegetables, fish, whole grains, and olive oil, should be promoted, and pro-inflammatory food, such as red meat, processed food, and animal oils, should be avoided. Further well-designed prospective trials are needed to strengthen the evidence of the associations between the dietary inflammatory potential and mental health.

## Supplementary Information

**Additional file1**: PRISMA checklist.

**Additional file 2**: References of studies excluded in the meta-analysis.

**Additional file 3**: Methodological quality of the included studies.

## Data Availability

All data generated or analyzed during this study are included in this published article.

## Abbreviations

HR: Hazard ratio
OR: Odds ratio
RR: Relative risk
PUFAs: Polyunsaturated fatty acids
SFA: Saturated fatty acids
PRISMA: Preferred Reporting Items for Systematic Reviews and Meta-Analyses
NOS: Newcastle–Ottawa Scale
DII: Dietary Inflammation Index
AHRQ: Agency for Healthcare Research and Quality
E-DII/ADII/FDII: Modified DII
CES-D: The Centre for Epidemiological Studies Depression Scale
PHQ-9: Patient Health Questionnaire 9
DASS-21: Depression Anxiety and Stress Scales
HADS: The Hospital Anxiety and Depression Scale
