# Supplementary material for: Dietary inflammatory potential and the incidence of depression and anxiety: a meta-analysis
Source: J Health Popul Nutr. 2022 May 28;41:24. doi: 10.1186/s41043-022-00303-z (PMC9148520; doi:10.1186/s41043-022-00303-z)
Supplement: Supplementary file 2 — Additional file 2: References of studies excluded in the meta-analysis. [file 41043_2022_303_MOESM2_ESM.doc]

**Supplementary Table 2.** **References of studies excluded in the meta-analysis**

| **not provide sufficient data (N=4)** |
| --- |

1. Ghazizadeh H, Yaghooti-Khorasani M, Asadi Z, et al. Association between Dietary Inflammatory Index (DII) and depression and anxiety in the Mashhad Stroke and Heart Atherosclerotic Disorder (MASHAD) Study population. BMC Psychiatry. 2020; 20(1).

2. Milajerdi A, Keshteli A H, Afshar H, et al. Dietary total antioxidant capacity in relation to depression and anxiety in Iranian adults. Nutrition. 2019; 65:85-90

3. Weng T T, Hao J H, Qian Q W, et al. Is there any relationship between dietary patterns and depression and anxiety in Chinese adolescents?. Public Health Nutr. 2012; 15(4):673-82

4.Saeidlou S N, Kiani A, Ayremlou P. Association between Dietary Patterns and Major Depression in Adult Females: A Case-Control Study. Journal of Research in Health Sciences. 2021; 21(1):e00506.

| **Review articles (N=5)** |
| --- |

5.Ljungberg T, Bondza E, Lethin, C. Evidence of the Importance of Dietary Habits Regarding Depressive Symptoms and Depression. Int J Environ Res Public Health. 2020; 02:17(5)

6.Silva D, Cobucci R N, Gonalves A K, et al. Systematic review of the association between dietary patterns and perinatal anxiety and depression. BMC Pregnancy and Childbirth. 2019; 19(1) :212

7.Kris-Etherton PM, Petersen KS, Hibbeln JR, Woodward-Lopez G. Nutrition and behavioral health disorders: depression and anxiety. Nutr Rev. 2021; 79(3) :247-260

8.Khalid S, Williams C M, Reynolds S A. Is there an association between diet and depression in children and adolescents? A systematic review. British Journal of Nutrition. 2016; 116(12):2097-2108.

9.Hoffmann K, Emons B, Brunnhuber S, et al. The Role of Dietary Supplements in Depression and Anxiety-A Narrative Review. Pharmacopsychiatry. 2019; 52(06):261-279.

| **Risk estimates not available (N=2)** |
| --- |

10.Burrows K, Stewart J L, Antonacci C, et al. Association of Poorer Dietary Quality and Higher Dietary Inflammation with Greater Symptom Severity in Depressed Individuals with Appetite Loss. Journal of Affective Disorders. 2019; 263.

11.Swann O G, Michelle K, Monique B, et al. Dietary fiber and its associations with depression and inflammation. Nutr Rev. 2020; 78(5):394 -411

| **Full-text not available (N=1)** |
| --- |

12.Kawada T. Dietary Inflammatory Index and psychological disorders. Clinical Nutrition. 2019; 39(1).

| **Not observational study (N=4)** |
| --- |

13.Siavash, Fazelian, Reza, et al. Effect of Vitamin D Supplement on Mood Status and Inflammation in Vitamin D Deficient Type 2 Diabetic Women with Anxiety: A Randomized Clinical Trial. International journal of preventive medicine. 2019; 10:17-17.

14.Oliver-Baxter J M, Whitford H S, Turnbull D A , et al. Effects of vitamin supplementation on inflammatory markers and psychological wellbeing among distressed women:a randomized controlled trial. J Integr Med. 2018; 16(5):322-328

15.Bourdel-Marchasson I, Ostan R, Regueme S C, et al. Quality of Life: Psychological Symptoms—Effects of a 2-Month Healthy Diet and Nutraceutical Intervention; A Randomized, Open-Label Intervention Trial (RISTOMED). Nutrients. 2020; 12(3).

16.Cauffield J S, Forbes H J. Dietary supplements used in the treatment of depression, anxiety, and sleep disorders. Lippincotts Prim Care Pract. 1999; 3(3):290-304

| **No data on dietary inflammation index or blood inflammation levels were provided (N=10)** |
| --- |

17.Oliver-Baxter J M, Whitford H S, Turnbull D A, et al. Effects of vitamin supplementation on inflammatory markers and psychological wellbeing among distressed women:a randomized controlled trial. Arch Iran Med. 2017; 20(2):108-112

18.Wen J, Mo M, Li M, et al. The relationship of dietary diversity score with depression and anxiety among prenatal and post-partum women. Journal of Obstetrics and Gynaecology Research. 2018; 44(10):1929-1936

19.Rahmani, Jamal, Milajerdi, et al. Association of the Alternative Healthy Eating Index (AHEI-2010) with depression, stress and anxiety among Iranian military personnel. J R Army Med Corps. 2018; 164(2):87-91

20.Noguchi R, Hiraoka M, Watanabe Y, et al. Relationship between Dietary Patterns and Depressive Symptoms: Difference by Gender, and Unipolar and Bipolar Depression. Journal of Nutritional Science & Vitaminology. 2013; 59(2):115-122.

21.Poorrezaeian M, Siassi F, Milajerdi A, et al. Depression is related to dietary diversity score in women: a cross-sectional study from a developing country. Annals of General Psychiatry. 2017; 16(1):39.

22.Daneshzad E, Keshavarz S A, Qorbani M, et al. Association of Dietary Acid Load and Plant-based Diet Index with Sleep, Stress, Anxiety, and Depression in Diabetic women. British Journal Of Nutrition. 2019; 123(8):1-32.

23.Saneei P, Hajishafiee M, Keshteli A H, et al. Adherence to Alternative Healthy Eating Index in relation to depression and anxiety in Iranian adults. British Journal of Nutrition. 2016:335-342.

24.Park S J, Kim M S, Lee H J. The association between dietary pattern and depression in middle-aged Korean adults. Nutr Res Pract. 2019; 13(4) :316-322.

25.Kim C S, Byeon S, Shin D M. Sources of Dietary Fiber Are Differently Associated with Prevalence of Depression. Nutrients. 2020; 12(9)

26.Dipnall JF, Pasco JA, Meyer D, et al. The association between dietary patterns, diabetes and depression. J Affect Disord. 2015; 174:215-224.
