# Supplementary material for: Dietary inflammatory potential and the incidence of depression and anxiety: a meta-analysis
Source: J Health Popul Nutr. 2022 May 28;41:24. doi: 10.1186/s41043-022-00303-z (PMC9148520; doi:10.1186/s41043-022-00303-z)
Supplement: Supplementary file 3 — Additional file 3: Methodological quality of the included studies. [file 41043_2022_303_MOESM3_ESM.docx]

Supplementary Table s3 a Methodological quality of cohort studies included in the meta-analysis.

| Cohort studies^a^ | Selection | | | | Comparability^b^ | Outcome | | | score |
| --- | --- | --- | --- | --- | --- | --- | --- | --- | --- |
|  | Representativenese of the exposed cohort | Selection of  the unexposed cohort | Ascertainment of exposure | Outcome of interest not present at start of study | Control for important factor or additional factor | Outcome assessment | Follow-up long enough for outcome to occur^c^ | Adequacy of follow-up of cohorts^d^ |  |
| Adjibade et al., 2019 [18] | * | * | * | * | ** | * | * | * | 9 |
| Akbaraly et al., 2016 [19] |  | * | * | * | ** | * | * |  | 7 |
| Lucas et al., 2014 [23] | * | * | * | * | * | * | * | * | 8 |
| Sanchez-Villages et al., 2015 [27] |  | * |  | * | ** | * | * | * | 7 |
| Shivappa et al., 2016 [29] | * | * | * | * | ** | * | * |  | 8 |
| Shivappa et al., 2018 [30] | * | * | * | * | ** | * | * |  | 8 |
| Vermeulen et al., 2018 [31] | * | * | * | * | * | * | * | * | 8 |
| Adjibade et al., 2017 [33] | * | * | * | * | * | * | * |  | 7 |

^a^A study can be awarded Q19 a maximum of one star for each item except the item “Comparability.”

^b^A maximum of two stars can be awarded for this item. Studies controlling for or matching by age, gender received one star while studies additionally controlling for other important confounders received an additional star.

^c^A cohort study with a follow-up time of more than 5 years was assigned one star.

^d^A cohort study with a follow-up rate of more than 90% was assigned one star

Supplementary Table s3 b Methodological quality of cross-sectional studies included in the meta-analysis.

| Study  Item | Açik et al., 2019 [17] | Bergmans et al., 2017 [20] | Haghighatdoost et al., 2019 [21] | Jorgensen et al., 2018 [22] | Phillips et al., 2018 [24] | Salari-Moghaddam et al., 2019 [25] | Salari-Moghaddam et al., 2019 [26] | Shivappa et al., 2018 [28] | Wirth et al., 2017 [32] |
| --- | --- | --- | --- | --- | --- | --- | --- | --- | --- |
| Define the source of information (survey, record review) | Yes | Yes | Yes | Yes | Yes | Yes | Yes | Yes | Yes |
| List inclusion and exclusion criteria for exposed and unexposed subjects (cases and controls) or refer to previous publications | Yes | Yes | Yes | Yes | Yes | Yes | Yes | Yes | Yes |
| Indicate time period used for identifying patients | Yes | Yes | Yes | Yes | Yes | Yes | Yes | Yes | Yes |
| Indicate whether or not subjects were consecutive if not population-based | Yes | Yes | Yes | Yes | Yes | Yes | Yes | Yes | Yes |
| Indicate if evaluators of subjective components of study were masked to other aspects of the status of the participants | No | No | No | No | No | No | No | No | No |
| Describe any assessments undertaken for quality assurance purposes (e.g., test/retest of primary outcome measurements) | Unclear | No | No | Unclear | No | No | No | Unclear | No |
| Explain any patient exclusions from analysis | Yes | Yes | Yes | Yes | Yes | Yes | Yes | Yes | Yes |
| Describe how confounding was assessed and/or controlled | Yes | Yes | Yes | Yes | Yes | Yes | Yes | Yes | Yes |
| lf applicable, explain how missing data were handled in the analysis | No | No | Yes | No | Yes | No | No | Yes | Yes |
| Summarize patient response rates and completeness of data collection | Yes | Yes | Yes | Yes | Yes | Yes | Yes | Yes | Yes |
| Clarify what follow-up, if any, was expected and the percentage of patients for which incomplete data or follow-up was obtained | No | Unclear | No | No | No | No | No | No | Unclear |
| Quality score | 7 | 7 | 8 | 7 | 8 | 7 | 7 | 8 | 8 |
